# Predictive Factors of Neoadjuvant Chemotherapy Response in Breast Cancer Validated by Three Anatomopathological Scores: Residual Cancer Burden, Chevallier System, and Tumor-Infiltrating Lymphocytes

**DOI:** 10.7759/cureus.59391

**Published:** 2024-04-30

**Authors:** Cristian N Popa, Rodica Bîrlă, Dinu Daniela, Cristina Iosif, Evelina Chirita, Ioan Nicolae Mateș

**Affiliations:** 1 Faculty of Medicine, University of Medicine and Pharmacy "Carol Davila", Bucharest, ROU; 2 Department of General Surgery I, "Sf. Maria" Clinical Hospital, Bucharest, ROU; 3 Department of General Surgery I, “Sf. Maria” Clinical Hospital, Bucharest, ROU; 4 Department of Pathology, "Sf. Maria" Clinical Hospital, Bucharest, ROU; 5 Department of Oncology, "Sf. Maria" Clinical Hospital, Bucharest, ROU

**Keywords:** chevallier system, tumor-infiltrating lymphocytes score, residual cancer burden score, predictive factors, neoadjuvant chemotherapy, breast cancer

## Abstract

Introduction: The aim of this retrospective, observational, descriptive study was to identify predictors of response to neoadjuvant therapy in breast cancer patients and to validate them using three anatomopathological scores, such as residual cancer burden (RCB) score, Chevallier system, and tumor-infiltrating lymphocytes (TIL) score.

Materials and methods: We conducted a study on 88 female patients aged 37 to 78 years with confirmed breast cancer who were indicated for neoadjuvant chemotherapy. We analyzed different individual variables (such as age, menarche, and menopause), clinical/imaging characteristics of the breast tumor and axillary nodes, immunohistochemical biomarkers (such as ER/PR/HER2 and Ki67), and histopathological features (such as subtype and grading) in relation to three anatomopathological scores calculated based on the surgical resection specimen.

Results: The percentage of patients who could have benefited from conservative surgery increased from 6% at the time of diagnosis to 20% post-primary systemic therapy (PST). Age under 49 (p = 0.01), premenopausal status (p < 0.01), no special type (NST) (p = 0.04), high Ki67 (p < 0.01), triple-negative breast cancer (TNBC) (p = 0.02) are positive predictive factors of neoadjuvant therapy, while lobular/mixt carcinoma-type (p = 0.04), luminal A (p = 0.01), positive lymph node (p < 0.01), and low differentiation grade (p = 0.01) are negative predictive factors for the response to PST.

Conclusion: There is a strong correlation between the RCB score and the Chevallier system for quantifying the response to PST, with most predictive factors being confirmed through appropriate statistical analysis for both. TIL score values correlated with only some of the predictors, most likely due to the importance of calculating this score on both biopsy specimens at diagnosis and resection specimens after chemotherapy.

## Introduction

Breast cancer is one of the three most common types of cancer worldwide, and the multimodal approach is the gold standard in treating this type of neoplasm. Currently, primary surgical resection, skipping neoadjuvant chemotherapy (ChT), is only performed in selected cases based on the molecular subtype and loco-regional characteristics of the tumor. The response to primary systemic therapy (PST) is a major decision-making factor in selecting patients who could benefit from breast-conserving surgery (BCS) and those who have a definite indication for radical surgery, simultaneously serving as an important and independent prognostic factor for recurrence and survival. The response to PST can be quantified through clinical and imaging methods as well as anatomopathological methods using data obtained from the resection specimen, the latter being considered the optimal method for evaluating this response [1-3]. Residual Cancer Burden (RCB) score, tumor-infiltrating lymphocytes (TIL) score, and Chevallier score are validated scoring systems based on anatomopathological information from the surgical resection specimen, each of them demonstrating predictive and prognostic value in breast cancers. However, there has been no comparison and, consequently, no correlation between these scores to date [4-6].

The objective of this retrospective, observational, descriptive study was to identify predictive factors for the response to neoadjuvant treatment in breast cancer patients to increase the number of cases eligible for BCS and also validate them, not only through a single anatomopathological method but through all three methods mentioned above.

The Chevallier system is a histopathological grading system used to assess the response of breast cancer to neoadjuvant ChT, which was developed in 1993 and used since then. This system categorizes the degree of tumor regression based on the proportion of residual cancer cells and fibrosis observed in the post-treatment surgical specimen. The Chevallier system typically grades responses into three categories; they are pCR (pathological complete response): the disappearance of the entire tumor from the breast with negative axillary lymph nodes (ypT0 ypN0); pPR (pathological partial response): the presence of invasive carcinoma with stromal alterations; and pNR (pathological no response): minimal changes to the primary tumor [7].

The RCB score is a newer validated method for assessing response to neoadjuvant therapy and represents an important and independent prognostic factor. The RCB index is calculated based on the anatomopathological data of the surgical resection specimen. It estimates the area containing residual malignant cells, marks this area on each slide analyzed microscopically, and finally reconstructs it to determine the zone represented by the tumor itself. Essentially, it mathematically analyzes certain characteristics of the anatomopathological specimen, such as the size of the primary tumor and the percentage of invasive versus in situ tumors, in addition to analyzing the number of positive lymph nodes and the size of the largest positive lymph node [5]. The RCB online calculator developed by the MD Anderson Cancer Center at the University of Texas calculates a response index, where 0 is equivalent to pCR, RCB-1 (minimal residual disease), RCB-II (moderate residual disease), and RCB-III (extensive) [8].

TILs can be evaluated using tissue obtained from core biopsies before neoadjuvant treatment or tissue from specimens obtained after surgical resection before adjuvant treatment [9]. Recommendations for calculating the TIL score in breast cancer include reporting TILs as a percentage of the stromal compartment, specifically the percentage of mononuclear inflammatory cells in the entire stromal area, respecting the tumor margins and excluding normal tissue or DCIS as well as necrotic or hyalinized areas. At this time, there is no clear recommendation for immunohistochemistry to determine lymphocyte subtypes, and their clinical significance is still under research [10,11]. In this study, the TIL score could only be estimated on surgical resection specimens and not on those obtained through diagnostic biopsies at the time of diagnosis (diagnostic biopsies performed in multiple centers, whose slides could not be subsequently obtained). Depending on the percentage of lymphocytes, the TIL score is divided into TIL 0 (for percentages of 1%-10%), TIL 1 (10%-40%), TIL 2 (40%-70%), and TIL 3 (70%-90%).

## Materials and methods

From July 2018 to December 2022, data were collected on 240 breast cancer cases treated surgically at the Department of General Surgery I, “Sf. Maria” Clinical Hospital, Bucharest. This retrospective, observational, descriptive study included only cases for which clinical data (primary tumor status and axillary lymph nodes), imaging data (ultrasonography [US]/mammography/magnetic resonance imaging [MRI]) performed before and after neoadjuvant PST, and diagnostic data (anatomopathological and immunohistochemical [IHC]) were complete as well as those with an indication for neoadjuvant ChT. Cases with distant metastases or severe associated pathologies that contraindicated ChT, those without an indication for neoadjuvant ChT, and those for which anatomopathological slides with surgical resection specimens could not be accessed (CISH/FISH performed in other centers) were excluded. Chest computed tomography (CT), US, abdominal CT or MRI, and bone scintigraphy were performed on all patients with clinically/imaging-positive axillary lymph nodes and tumors over 5 cm (64 cases).

The diagnosis of breast cancer was made on the tumor tissue obtained through core needle biopsy/tru-cut and analyzed for tumor type (no special type [NST]/lobular/mixed), tumor grade-Nottingham Score [12], and the presence of vascular and/or perineural invasion. Histopathological classification was done according to the World Health Organization. Through immunohistochemistry (IHC), the analysis of hormonal receptors (ER and PR), HER2, and the cellular proliferation marker Ki67 was performed on the biopsy specimen at the time of diagnosis.

Clinical findings were integrated with imaging (mammography or US) to determine the size of the primary tumor (T) and the presence or absence of regional lymph node metastases (N). Additionally, in the database, the age of the patients at the time of diagnosis, menopausal status, and the localization of the primary tumor (right breast/left breast and quadrant) have been entered.

ChT was given every 21 days/four cycles for AC regimen (doxorubicin 60 mg/m^2^ plus cyclophosphamide 600 mg/m^2^), EC regimen (epirubicin 60 mg-90 mg/m^2^), FEC (5-fluorouracil 600 mg/m^2^ days 1 and 8 plus epirubicin 60 m^2^mg-90 mg/m^2^ plus cyclophosphamide 600 mg/m^2^), FAC (5-fluorouracil 600 mg/m^2^ days 1 and 8 plus doxorubicin 60 mg/m^2^ plus cyclophosphamide 600 mg/m^2^), and TAC regimen (docetaxel 75 mg/m^2^ plus doxorubicin 50 mg/m^2^ plus cyclophosphamide 500 mg/m^2^). CMF regimen (cyclophosphamide 100 mg/m^2^ days 1-14, methotrexate 40 mg/m^2^ days 1 and 8 plus 5-fluorouracil 600 mg/m^2^ days 1 and 8) was given six cycles every 28 days. Paclitaxel (PTX) 80 mg/m^2^ was administered after AC/EC or CMF regimens, weekly, for 12 weeks. All HER2+ cases received different doses of trastuzumab depending on body weight.

After finishing the personalized ChT regimens, surgical treatment was performed consisting of either sectoral resection/lumpectomy and partial axillary lymph node dissection or Madden-modified radical mastectomy. Response to neoadjuvant ChT was assessed based on the surgical resection specimen by calculating the three previously mentioned scores: Chevallier, RCB, and TIL.

All characteristics of each patient previously mentioned have been statistically analyzed in relation to the three aforementioned anatomopathological scores. For statistical analysis, the likelihood ratio test was used in univariate analysis. In cases where p-values (p_value) were compared, they were adjusted using the Bonferroni method. The Chi-square test was used both for analyzing categorical data (to assess the association or differences between variable categories) and for estimating the necessary sample size for the study and statistical power. Statistical analysis was conducted using SPSS software, version 23.0 (IBM Corp., Armonk, NY), and p < 0.05 was considered statistically significant.

## Results

The final cohort consisted of 88 female patients aged between 37 and 78 years. The predominant histopathological type was invasive carcinoma NST in 70 cases (79.5%); lobular and mixed types were much less common, found in 15 and three cases, respectively. Regarding tumor grading, there were five cases classified as grade 1 (G1: well-differentiated), 58 cases classified as G2 (moderately differentiated), and 25 cases classified as G3 (poorly differentiated). Regarding the size of the primary tumor, most of them fell between 2 and 5 cm (56 cases), with 14 cases having dimensions over 5 cm, and 18 cases with tumors ≤ 2 cm. There were 22 cases (25%) of multiple breast tumors. A total of 65 patients had clinically positive axillary lymph nodes at the time of diagnosis. According to TNM (tumor size, lymph nodes affected, metastases) classification, cases were classified into stages II (A-14 cases and B-37 cases) and III (A-17 cases and B-19 cases), with only one case in stage I [12].

In the analyzed cohort, there were 66 estrogen receptor-positive (ER+) cases and 54 progesterone receptor-positive (PR+) cases. The expression of the HER2 protein (gene amplification/overexpression) was confirmed in 17 cases (6 within the HER2+ subtype and 11 in luminal B/HER2+) [13]. Ki67 status was also determined using immunohistochemistry and analyzed as a percentage, resulting in 20 cases with values under 10%, 29 cases between 10% and 30%, and the majority (39 cases) with values over 30% [14]. While classifying the cases into immunohistochemical subtypes, it was observed that most patients fell into the luminal B subtype with 48 cases (HER2- 37 cases, HER2+ 11 cases), followed by luminal A with 19 cases, basal with 15 cases, and HER2+ with six cases.

A doxorubicin/cyclophosphamide (AC) regimen was used in most of the cases (33 patients), followed by epirubicin/cyclophosphamide (EC) in 28 cases; seven cases received 5-fluorouracil/epirubicin/cyclophosphamide (FEC), six cases received 5-fluorouracil/doxorubicin/cyclophosphamide (FAC), and three patients received docetaxel/doxorubicin/cyclophosphamide (TAC). Cyclophosphamide/methotrexate/5-fluorouracil (CMF) regimen was given to 11 patients. PTX was administered in 68 cases after AC/EC or CMF regimens, and all 17 HER2+ cases received different doses of trastuzumab [15].

After conducting a clinical and post-PST imaging assessment, only 18 cases had tumors ≤ 20 mm. Among these, two cases with multiple tumors could not undergo BCS, four cases had tumors involving the skin, and two cases involved patients who opted for total mastectomy. In the analyzed cohort, 23 patients had clinically negative axillary lymph nodes post-PST (cN0). Of these, 10 cases did not require sentinel lymph node biopsy (SLNB) due to the size of the primary tumor (T3/T4), seven either did not have access to this method at the time of diagnosis or refused post-surgical tangential radiation therapy, opting for complete lymph node clearance, and six were reclassified as +cN after imaging examination. Therefore, 10 cases were treated with sectoral resection/lumpectomy and partial axillary lymph node dissection - removal of lymph node stations I and II (Berg), and 78 cases underwent modified radical mastectomy of the Madden type [16]. Practically, only 11.3% of patients underwent conservative surgical treatment, even though the percentage of patients who could have benefited from BCS was approximately 20% post-PST. Practically, the percentage of patients who could have benefited from conservative surgery increased from 6% at the time of diagnosis to 20% post-PST.

The Chevallier system

Among the cohort of 88 patients, 16 achieved pCR after neoadjuvant ChT, while the remaining 72 had pPR, with no patients falling into the pNR category. Patients with pCR were observed in all age groups, but there were statistically significant differences in the 40-49 age subgroup, with 43.8% achieving pCR compared to 15.3% with pPR, and in the 60-69 age subgroup, with 12.5% achieving pCR compared to 38.9% with pPR (p = 0.01). In close association with age, significant differences were also obtained in terms of menopausal status, specifically, half of the patients who achieved pCR were postmenopausal (50%) compared to 88.9% of patients with partial response to neoadjuvant ChT (p < 0.01).

Out of the 16 pCR cases, all belonged to the ductal type, with statistical differences observed, specifically in the ductal subgroup with percentage differences of 100% pCR compared to 75% pPR and in the lobular subgroup with 0% pCR compared to 20.8% pPR (p = 0.04).

Among the 16 patients with pCR, the majority (nine cases) fell into the luminal B subtype, five into the basal subtype, and two into the HER2- subtype, with no cases falling into the luminal A subtype. Furthermore, it can be observed that 80.6% of patients with pPR belonged to the luminal A and luminal B subgroups. Significant results were obtained in the luminal A subgroup, with differences between 0% pCR and 26.4% pPR (p = 0.01).

Regarding the Ki67 status, relevant results were obtained in the subgroup with ≤10%, with 0% pCR compared to 27.8% pPR (p < 0.01), suggesting a weaker response to neoadjuvant ChT in tumors with low Ki67 percentages, making Ki67 status a potential negative prognostic factor for the response to PST. In relation to tumor grading, significant results were obtained in the G1 subgroup, with 18.8% of cases achieving pCR compared to 2.8% with pPR, and in the G2 subgroup, with 37.5% achieving pCR compared to 72.2% with pPR (p = 0.01). These data suggest a favorable response to ChT in well-differentiated tumors, with response rates significantly decreasing as the degree of differentiation decreases. Therefore, grading is a predictive factor for the response to PST, being positive for G1 and negative for G2. Part of the results are presented in Table 1.

**Table 1:** Predictive factors of response to neoadjuvant chemotherapy reported by the Chevallier system pR: Pathological response; pCR: Pathological complete response; pPR: Pathological partial response; HER2: Human epidermal growth factor receptor 2; IHC: Immunohistochemistry; G: Grading.

	pR = pCR (16)	pR = pPR (72)	p-value (test)
*Age (years)*			0.010971 (likelihood ratio)
<40	2/16 (12.5%)	1/72 (1.4%)	
40-49	7/16 (43.8%)	11/72 (15.3%)	
50-59	2/16 (12.5%)	22/72 (30.6%)	
60-69	2/16 (12.5%)	28/72 (38.9%)	
70-79	3/16 (18.8%)	10/72 (13.9%)	
Menopausal status	8/16 (50%)	64/72 (88.9%)	0.001190 (Fisher's exact test)
HER2	6/16 (37.5%)	11/72 (15.3%)	0.073621 (Fisher's exact test)
*Ki67*			0.008755 (likelihood ratio)
≤10	0/16 (0%)	20/72 (27.8%)	
10-30	6/16 (37.5%)	23/72 (31.9%)	
≥30	10/16 (62.5%)	29/72 (40.3%)	
*IHC*			0.015544 (likelihood ratio)
Basal	5/16 (31.3%)	10/72 (13.9%)	
Her2	2/16 (12.5%)	4/72 (5.6%)	
Luminal A	0/16 (0%)	19/72 (26.4%)	
Luminal B	9/16 (56.3%)	39/72 (54.2%)	
*G*			0.014337 (likelihood ratio)
1	3/16 (18.8%)	2/72 (2.8%)	
2	6/16 (37.5%)	52/72 (72.2%)	
3	7/16 (43.8%)	18/72 (25%)	
	pR = pCR (16)	pR = pPR (72)	p-value (test)

RCB score

Following the anatomopathological analysis of the 88 cases, only 14 patients fell into the RCB=0 category, indicating a complete pathological response to neoadjuvant ChT. Four patients had a very good response, categorized as RCB-I, while 23 patients had a moderate response (RCB-II). A significant percentage of 53.4% (47 patients) had minimal or no response to treatment, resulting in RCB=III. All 14 cases with pCR (pathological complete response) were invasive NST carcinomas. Half of these cases were in the age group of 40-49 years, and 57.1% were luminal B subtypes. There was only one case of multiple tumors, and no cases were categorized as cN2 (only cN0 or cN1). In the neoadjuvant therapy regimen, 78.6% of them received paclitaxel.

Regarding the response to neoadjuvant therapy, it was observed that 64.3% of patients under the age of 49 achieved a complete response to ChT. In contrast, 87.3% of patients with RCB-III were in the age range of 50-70 years, with statistical differences in this regard (p = 0.01). Specifically, in the age group of <40 years, 25% of patients fell into the RCB-I category, with none in RCB-III. In the 40-49 age group, 50% had a complete response to ChT, and only 12.8% (six patients) fell into the RCB-III category. Additionally, relevant results (p < 0.01) were obtained related to menopausal status, specifically between the RCB-0 and RCB-III groups. Only six out of 14 patients (42.9%) with a complete response to ChT were postmenopausal, compared to 42 out of 47 (89.4%) in the postmenopausal category with RCB-III.

Concerning the size of the primary tumors, 56 cases (63.6%) were in the range of 2-5 cm, 18 cases were ≤2 cm, and 14 cases were over 5 cm. When comparing the response to neoadjuvant therapy in these three size subgroups, differences were observed (p < 0.01) in relation to the RCB score. In the RCB-0 and RCB-I groups, the predominating tumor size range was ≤2 cm and 2-5 cm, while in the RCB-II and RCB-III groups, the 2-5 cm and >5 cm size ranges were more predominant. Specifically, within the ≤2 cm size range, RCB-I and RCB-II had response rates of 75% and 34.8%, respectively, compared to RCB-III (6.4%). In the 2-5 cm size range, RCB-III had a significantly higher response rate (78.7%) compared to RCB-II (43.5%).

Related to the axillary lymph node status, in the RCB-III subgroup, 87.2% of patients had +N status (positive axillary lymph nodes), compared to only 43.5% (10 out of 23 patients) in the RCB-II subgroup (p < 0.01).

Of all cases, 66 were ER+, and 54 cases were PR+. A percentage of 62.1% of ER+ cancers and 62.9% of PR+ cancers had a significant residual disease and were categorized as RCB-III. Although there were significant differences, precise statistical distinctions between RCB groups could not be determined. However, this suggests a poor response to ChT in hormone receptor-positive cancers. It was observed that 50% of all cases classified as RCB-I belonged to the basal immunohistochemical subtype, with only 6.4% of this subtype in the RCB-III group.

Within our cohort, differences were observed (p < 0.01) in the subgroups of G1, with 21.4% of patients classified as RCB-0 compared to 0% (no patients) in the RCB-III subgroup. In the G2 subgroup, 28.6% of patients were classified as RCB-II, and 80.9% were classified as RCB-III, suggesting a decrease in the response rate to neoadjuvant ChT with an increase in grading and a decrease in differentiation.

In the analysis of all neoadjuvant ChT regimens about the Anderson score, no significant results were obtained, likely due to the relatively large number of therapeutic regimens (8) compared to the number of cases (88). It is clear that an expansion of the cohort is necessary for a coherent statistical analysis.

Following the histopathological analysis of the surgical resection specimens from all 88 patients, it was observed that 35 patients had negative axillary lymph nodes on the specimen (N0), with all 14 cases categorized as RCB-0 and only one case (2.1%) categorized as RCB-III (p < 0.01). The ypTNM staging (post-neoadjuvant therapy resection specimen) highlights a clear association between early stages and a better response to neoadjuvant ChT, with the response rate decreasing as the tumor stage increases (p < 0.01). Statistically significant results (p < 0.01) were also obtained regarding perineural and perivascular invasion. Specifically, none of the patients classified as RCB-0 had perineural invasion, whereas 83% (39 out of 47 patients) classified as RCB-III had perineural invasion. As for perivascular invasion, it was detected in only one patient out of the 14 classified as RCB-0, compared to 95.7% (45 out of 47 patients) classified as RCB-III. Part of the results are presented in Table 2.

**Table 2:** Predictive factors of response to neoadjuvant chemotherapy reported by the residual cancer burden score RCB: Residual cancer burden; IHC: Immunohistochemistry; T: Tumor; N: Nodes; G: Grading.

	RCB = 0	RCB = I	RCB = II	RCB = III	p-value (test)
*Age (years)*					0.012538 (likelihood ratio)
<40	2/14 (14.3%)	1/4 (25%)	0/23 (0%)	0/47 (0%)	
40-49	7/14 (50%)	1/4 (25%)	4/23 (17.4%)	6/47 (12.8%)	
50-59	2/14 (14.3%)	1/4 (25%)	4/23 (17.4%)	17/47 (36.2%)	
60-69	2/14 (14.3%)	1/4 (25%)	12/23 (52.2%)	15/47 (31.9%)	
70-79	1/14 (7.1%)	0/4 (0%)	3/23 (13%)	9/47 (19.1%)	
Menopausal status	6/14 (42.9%)	3/4 (75%)	21/23 (91.3%)	42/47 (89.4%)	0.002428 (likelihood ratio)
*Ki67*					0.051950 (likelihood ratio)
≤10	0/14 (0%)	0/4 (0%)	6/23 (26.1%)	14/47 (29.8%)	
10-30	6/14 (42.9%)	2/4 (50%)	5/23 (21.7%)	16/47 (34%)	
≥30	8/14 (57.1%)	2/4 (50%)	12/23 (52.2%)	17/47 (36.2%)	
*IHC*					0.027918 (likelihood ratio)
Basal	4/14 (28.6%)	2/4 (50%)	6/23 (26.1%)	3/47 (6.4%)	
Her2	2/14 (14.3%)	0/4 (0%)	1/23 (4.3%)	3/47 (6.4%)	
Luminal A	0/14 (0%)	0/4 (0%)	5/23 (21.7%)	14/47 (29.8%)	
Luminal B	8/14 (57.1%)	2/4 (50%)	11/23 (47.8%)	27/47 (57.4%)	
*T*					0.005570 (likelihood ratio)
≤2	4/14 (28.6%)	3/4 (75%)	8/23 (34.8%)	3/47 (6.4%)	
2-5	8/14 (57.1%)	1/4 (25%)	10/23 (43.5%)	37/47 (78.7%)	
>5	2/14 (14.3%)	0/4 (0%)	5/23 (21.7%)	7/47 (14.9%)	
*N*					0.005570 (likelihood ratio)
N0	5/14 (35.7%)	0/4 (0%)	9/23 (39.1%)	3/47 (6.4%)	
N1	9/14 (64.3%)	4/4 (100%)	11/23 (47.8%)	40/47 (85.1%)	
N2	0/14 (0%)	0/4 (0%)	3/23 (13%)	4/47 (8.5%)	
*G*					0.001820 (likelihood ratio)
1	3/14 (21.4%)	1/4 (25%)	1/23 (4.3%)	0/47 (0%)	
2	4/14 (28.6%)	1/4 (25%)	15/23 (65.2%)	38/47 (80.9%)	
3	7/14 (50%)	2/4 (50%)	7/23 (30.4%)	9/47 (19.1%)	

TIL score

Regarding age groups, it was observed that all five cases with TIL 3 were over 50 years old, while 47.3% of cases with TIL 0 were between the ages of 40 and 49, without statistically proving the association between younger ages and low TIL scores (p = 0.06) within the cohort. Regarding menopausal status, relevant differences were observed between TIL 0 (with 63.2% of patients in post-menopause) compared to 100% in the TIL 2 subgroup (p < 0.01), suggesting an association between menopause and high TIL scores.

In terms of histopathological type, it was observed that all neoplasms with a TIL score of 0 belonged to the ductal type (invasive ductal carcinoma) (100%), while lobular or mixed subtypes were categorized as TIL 1 or TIL 2. Significant differences were obtained in the case of ductal histopathological type with 100% TIL 0 compared to 69.6% TIL 1 (p = 0.01), but the significance of this association in practice could not be clearly determined, especially in our cohort where 78.4% of cancers belonged to invasive ductal carcinoma. In the subgroups of immunohistochemical phenotypes, the distribution of cases based on the TIL score was relatively homogeneous, but there were no cases of TIL 0 in the luminal A subtype and no cases of TIL 3 in the HER2+ subtype.

The ki67 interval was analyzed in relation to the TIL score, and significant association (p < 0.01) was demonstrated between low ki67 values below 10% and higher TIL scores. In the subgroup with ki67 values below 10%, there were no patients with TIL 0, but 36.6% of them had TIL 1 scores. Furthermore, within the TIL 0 subgroup, all patients had high or intermediate values of this cell proliferation marker, suggesting a clear association between low TIL scores even after neoadjuvant ChT in patients with aggressive cancers and high ki67 values.

The distribution of ChT regimens within the cohort was discussed earlier, and statistically significant results (p < 0.01) were obtained between different ChT regimens concerning the TIL score. Within the AC subgroup (doxorubicin + cyclophosphamide), there are differences not only between 26.3% of cases categorized as TIL 0 and 70.6% categorized as TIL 2 but also between this latter percentage and 0% of cases categorized as TIL 3. This suggests the potential of this ChT regimen to categorize post-neoadjuvant therapy neoplasms into intermediate TIL groups (TIL 1 and TIL 2). Also, it can be mentioned that there is a relatively large number of HER2+ cases (pure HER2+ or within Luminal B) and triple-negative cases (a total of 17 out of 33 who received AC) that present intermediate TIL scores (1 or 2) after PST, with a favorable prognosis compared to those categorized as TIL 0. The same trend was observed for patients who received CMF, yielding statistically significant results of 0% in the TIL 0 and TIL 1 subgroups, compared to 60% (three out of five patients) categorized as TIL 3. This suggests an association of this ChT regimen with high TIL scores and a good response to this therapy, implicitly associated with a favorable prognosis. Furthermore, all five patients who received CMF had high TIL scores (TIL 2 and TIL 3) on the surgical resection specimen. No statistically significant results were obtained regarding neoadjuvant therapy with taxanes or trastuzumab.

In relation to the grade of differentiation (Nottingham score), relevant differences were observed (p = 0.03) in the G2 subgroup with 36.8% of cases categorized as TIL 0 compared to 78.3% of cases categorized as TIL 1. This indicates an association between this intermediate degree of differentiation and low TIL scores (75% of G2 cases categorized as TIL 0 and TIL 1). Part of the results are presented in Table 3.

**Table 3:** Predictive factors of response to neoadjuvant chemotherapy reported by the tumor-infiltrating lymphocytes score TIL: Tumor-infiltrating lymphocytes score; ChT: Chemotherapy; AC: Doxorubicin/cyclophosphamide; EC: Epirubicin/cyclophosphamide; FEC: 5-Fluorouracil/epirubicin/cyclophosphamide; FAC/FC: 5-Fluorouracil/doxorubicin/cyclophosphamide; TAC regimen: Docetaxel/doxorubicin/cyclophosphamide; CMF: Cyclophosphamide/methotrexate/5-fluorouracil; G: Grading.

	TIL = 0	TIL = 1	TIL = 2	TIL = 3	p-value (test)
*Age (years)*					0.066307 (likelihood ratio)
<40	1/19 (5.3%)	2/46 (4.3%)	0/17 (0%)	0/5 (0%)	
40-49	8/19 (42.1%)	10/46 (21.7%)	0/17 (0%)	0/5 (0%)	
50-59	2/19 (10.5%)	13/46 (28.3%)	6/17 (35.3%)	2/5 (40%)	
60-69	6/19 (31.6%)	13/46 (28.3%)	9/17 (52.9%)	2/5 (40%)	
70-79	2/19 (10.5%)	8/46 (17.4%)	2/17 (11.8%)	1/5 (20%)	
Menopausal status	12/19 (63.2%)	37/46 (80.4%)	17/17 (100%)	5/5 (100%)	0.005693 (likelihood ratio)
Multiple	1/19 (5.3%)	16/46 (34.8%)	5/17 (29.4%)	0/5 (0%)	0.014632 (likelihood ratio) Undetectable differences!
*Ki67*					0.003835 (likelihood ratio)
≤10	0/19 (0%)	15/46 (32.6%)	4/17 (23.5%)	0/5 (0%)	
10-30	9/19 (47.4%)	16/46 (34.8%)	3/17 (17.6%)	1/5 (20%)	
≥30	10/19 (52.6%)	15/46 (32.6%)	10/17 (58.8%)	4/5 (80%)	
*ChT*					0.001212 (likelihood ratio)
AC	5/19 (26.3%)	16/46 (34.8%)	12/17 (70.6%)	0/5 (0%)	
EC	8/19 (42.1%)	16/46 (34.8%)	3/17 (17.6%)	1/5 (20%)	
FAC	2/19 (10.5%)	3/46 (6.5%)	0/17 (0%)	1/5 (20%)	
FC	0/19 (0%)	5/46 (10.9%)	0/17 (0%)	0/5 (0%)	
FE	1/19 (5.3%)	0/46 (0%)	0/17 (0%)	0/5 (0%)	
FEC	2/19 (10.5%)	5/46 (10.9%)	0/17 (0%)	0/5 (0%)	
CMF	0/19 (0%)	0/46 (0%)	2/17 (11.8%)	3/5 (60%)	
TAC	1/19 (5.3%)	1/46 (2.2%)	0/17 (0%)	0/5 (0%)	
*G*					0.033987 (likelihood ratio)
1	3/19 (15.8%)	2/46 (4.3%)	0/17 (0%)	0/5 (0%)	
2	7/19 (36.8%)	36/46 (78.3%)	11/17 (64.7%)	3/5 (60%)	
3	9/19 (47.4%)	8/46 (17.4%)	6/17 (35.3%)	2/5 (40%)	
Vascular	5/19 (26.3%)	36/46 (78.3%)	17/17 (100%)	3/5 (60%)	0.000002 (likelihood ratio)
Perineural	3/19 (15.8%)	33/46 (71.7%)	13/17 (76.5%)	3/5 (60%)	0.000125 (likelihood ratio)

## Discussion

According to recent guidelines, almost all patients with breast cancer indicate neoadjuvant ChT, especially those with luminal B/HER2+, HER2+, and TNBC subtypes. The chosen surgical method, whether total mastectomy or partial mastectomy, largely depends on the response to PST. The evaluation of response to neoadjuvant therapy is preferably done through anatomopathological methods performed on the surgical resection specimen; however, there is currently no clear correlation between different anatomopathological scores. The specific objective of the study was to identify predictive factors for the response to neoadjuvant ChT and to validate them concurrently through the three most important anatomopathological scores at this time: RCB, TIL, and the Chevallier system. While the Chevallier system is already established for evaluating ChT response, the RCB score has recently demonstrated its utility, feasibility, and reproducibility in assessing pCR, and the TIL score is an important predictive and prognostic factor used in breast cancers and beyond.

The percentage of cases that could have benefited from BCS increased from 6% at the time of diagnosis to 20% post-PST, but for the reasons discussed earlier, only 11.3% underwent conservative surgery.

Age under 49 is associated with a favorable response to ChT. Due to the predominance of aggressive, hormone-negative breast cancer types in younger women (triple-negative, HER2+) [17], they tend to have a better response to neoadjuvant ChT, leading to a higher rate of pCR after PST. Although it is considered that premenopausal women have more aggressive forms of breast cancer and that premenopause interferes with hormonal therapy and its response, concerning only neoadjuvant ChT, it seems that younger women respond better than older postmenopausal women. This association has been demonstrated both in relation to the RCB score and the Chevallier system. So, premenopausal status may be a predictive factor for the response to neoadjuvant ChT, but this requires statistical adjustment based on the histopathological subtype of each individual case, an adjustment that can be performed in much larger patient cohorts [18].

Both in terms of RCB and pCR, invasive lobular carcinoma and mixed carcinoma respond less to neoadjuvant ChT compared to invasive ductal carcinomas but have a better prognosis [12]. These results suggest that the ductal histopathological type may be a positive predictive factor for the response to ChT, while the lobular type may be a negative predictive factor, with much lower response rates to ChT regimens.

Clinically/imaging-positive axillary lymph nodes represent a negative predictive factor for PST response. Within the cohort, axillary lymph node dissection (ALND) was performed in all patients. The clinical status of axillary lymph nodes is associated with large aggressive primary tumors based on grading and immunohistochemical characteristics as well as a high rate of breast cancer metastasis. Further studies are needed to adjust for primary tumor characteristics to confirm +N as a predictive factor for the response to neoadjuvant ChT [1,2].

Hormone receptor-positive (HR+) cancers (luminal A and luminal B/HER2-) respond favorably to ChT in percentages below 15%, with the utility of ChT in this patient category still under debate, while TNBC represents an aggressive subtype of breast cancer with reserved prognosis and a high mortality rate but with promising results regarding response to PST. These findings support existing literature indicating low rates of achieving pCR in the luminal A immunohistochemical subtype, which can be considered a negative predictive factor for the response to neoadjuvant ChT [2]. Although there was only one case of TNBC with RCB-0 (14.3%), the results obtained in this study align with those in the global literature, which mentions percentages of 36%-48% [17] for pathological complete response rates following neoadjuvant ChT, the only effective treatment option for TNBC.

A high Ki67 value seems to be a predictive factor for a favorable response to neoadjuvant ChT, but maintaining high values after PST remains an unfavorable prognostic factor for survival. The response rate to neoadjuvant ChT decreases with decreasing degree of tumor cell differentiation, practically with increasing Nottingham grading. Both associations have been validated through the RCB score and pCR.

TIL score could only be estimated on surgical resection specimens and not on those obtained by biopsy at the time of diagnosis, with the perspective of evaluating it as a predictive factor for adjuvant therapy and as a prognostic factor for survival. In the absence of a TIL score performed on biopsy tissue, no conclusions can be drawn regarding the change in TIL score post-neoadjuvant therapy to quantify the effect of ChT on this score. This limitation of the study does not allow for a concrete interpretation of the statistical results. However, an association has been observed between young age and implicitly premenopausal status with aggressive breast cancers, associating low TIL scores but still with favorable response to ChT according to the RCB score and pCR. Statistical results require adjustment according to the immunohistochemical subtype, knowing that the TIL score has predictive value for response to adjuvant/neoadjuvant ChT but proven prognostic value only in TNBC and HER2+ cases. TNBC and HER2+ cancers with intermediate TIL scores post-anthracycline-based neoadjuvant ChT are considered to have a favorable prognosis. This positive association was also observed in CMF regimens [6,8].

In this study, a clear association was observed between low TIL scores even after neoadjuvant ChT in patients with aggressive cancers characterized by high Ki67 values. In practice, cancers with intermediate and high grading are associated with low TIL scores, both characteristics suggesting aggressive forms, poor response to ChT, and a reserved prognosis [6].

## Conclusions

Most predictors of response to neoadjuvant ChT in breast cancer have been confirmed by both the RCB score and the Chevallier system, with statistically similar results for both anatomopathological methods. The TIL score, on the other hand, was correlated only with certain factors. A limitation of this study is the lack of information on the TIL score calculated on the biopsy specimen at the time of diagnosis, thus quantifying its post-ChT evolution.

Invasive lobular/mixt carcinoma-type HR+ cancers, clinically positive lymph node involvement at the time of diagnosis, and low differentiation grade are negative predictive factors for the response to PST, most of them confirmed by at least two anatomopathological scores. TNBC, a high Ki67 value, age under 49, and premenopausal status are predictive factors for a favorable response to PST. Additionally, TNBC and HER2+ cancers with intermediate TIL scores post-PST based on anthracyclines or CMF will have a favorable prognosis. On the other hand, a high Ki67 value after PST and young age are associated with a low TIL score, a reserved response to adjuvant therapy, and unfavorable prognostic for survival.
